# U-Shaped association between apolipoprotein A1 and serum uric acid levels in patients with osteoporotic fractures: a cross-sectional study

**DOI:** 10.3389/fendo.2025.1540879

**Published:** 2025-04-22

**Authors:** Guo-Ji Lin, Shao-Han Guo, Jia-Qi Liang, Ya-Qin Gong, Jian Jin, Chong Li, Ke Lu

**Affiliations:** ^1^ Department of Orthopedics, Affiliated Kunshan Hospital of Jiangsu University, Suzhou, Jiangsu, China; ^2^ Kunshan Biomedical Big Data Innovation Application Laboratory, Suzhou, Jiangsu, China; ^3^ Information Department, Affiliated Kunshan Hospital of Jiangsu University, Suzhou, Jiangsu, China; ^4^ Kunshan Municipal Health and Family Planning Information Center, Suzhou, Jiangsu, China

**Keywords:** apolipoprotein A1, uric acid, osteoporotic fractures, U-shaped relationship, uric acid trough

## Abstract

**Background:**

Lipid metabolism is closely linked to uric acid metabolism, with previous studies suggesting associations between lipid profiles to serum uric acid (SUA) levels. Apolipoprotein A1 (ApoA1), a key component in lipid metabolism and transport, may also be associated with SUA levels, though research in this area remains limited. This study aimed to investigate the independent association between ApoA1 levels and SUA in patients with osteoporotic fractures (OPF).

**Methods:**

This cross-sectional study included 2,108 OPF patients admitted to Kunshan Hospital, affiliated with Jiangsu University, from January 2017 to August 2023. Serum ApoA1 concentration was considered the exposure variable, and SUA concentration the outcome variable. Adjusted linear regression models and smooth curve fitting were employed to assess the relationship between ApoA1 and SUA. Nonlinear associations were examined using a generalized additive model (GAM), and a segmented regression method identified the inflection point. Univariate and stratified analyses were also performed.

**Results:**

Following adjustment for confounding covariates, a nonlinear relationship, U-shaped association was identified between serum ApoA1 and SUA concentrations, with an inflection point at 1.52 g/L. Estimated effects and 95% confidence intervals to the left and right of the inflection point were -55.20 (-75.57 to -34.82) and 77.33 (22.48 to 132.19), respectively.

**Conclusions:**

A U-shaped relationship between serum ApoA1 and SUA was identified in OPF patients. Based on these findings, we propose the concept of a “SUA trough” within the OPF population, additional research is required to explore the mechanism behind this association.

## Background

Osteoporotic fracture (OPF), also known as fragility fracture, commonly occur in the setting of osteoporosis or following minimal trauma, such as a fall from standing height, lifting objects, or even spontaneously without substantial injury (1, 2). OPFs have a profound impact on patients’ quality of life and independence. Globally, approximately one-third of women and one-fifth of men aged 50 and older are expected to experience fractures related to osteoporosis (3). In China alone, it is projected that by 2050, over 6 million new cases of OPF will arise, presenting a considerable healthcare challenge (4). As populations continue to age, the prevalence of OPF is anticipated to rise substantially (5).

Serum uric acid (SUA) is the final product of purine metabolism, derived from xanthine and hypoxanthine through the action of xanthine oxidoreductase (6). SUA has a complex role in the pathogenesis of osteoporosis, as it can promote bone loss by enhancing osteoclast activity and inflammatory responses (7). However, under certain conditions, SUA may also exert protective effects against bone loss (8). Although the upper limit of normal SUA is generally set at 420 µmol/L for males and 360 µmol/L for females (9), a study (10) demonstrated that even within the normal SUA range, the risk of cardiovascular disease increases progressively with higher SUA levels in individuals lacking traditional cardiovascular risk factors. Therefore, maintaining these prevention levels is of significant importance. Additionally, apolipoprotein A1 (ApoA1), the primary protein associated with high-density lipoprotein cholesterol (HDL-C), is essential for transporting cholesterol from peripheral tissues to the liver, thereby playing a vital role in lipid metabolism (11).

Previous studies have predominantly focused on the relationship between lipid profiles and SUA, revealing a positive correlation between SUA and the risk of hypertriglyceridemia (12). Lipid metabolism is intricately associated with apolipoproteins. Recent research has shown an inverse correlation between ApoA1 and SUA levels in healthy Chinese individuals (13), However, research on the relationship between apolipoproteins and SUA levels in patients with OPF remains limited. This study aims to further elucidate the relationship between ApoA1 and SUA levels, providing new insights into the complex interplay between lipid metabolism and uric acid metabolism. These insights may offer a fresh perspective on managing lipid and SUA levels in populations with OPF.

## Materials and methods

### Study design and subjects

**Figure 1:**
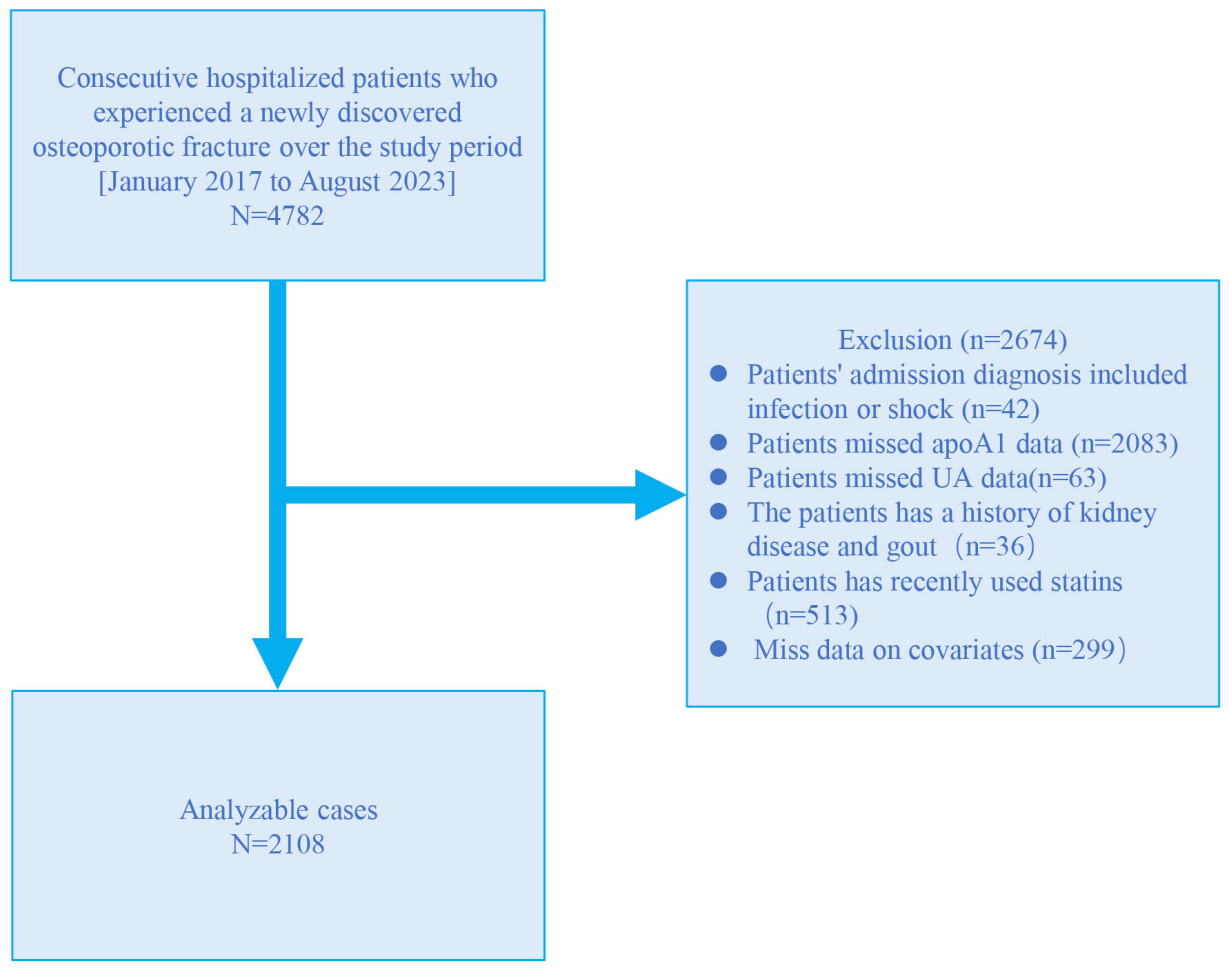
Study flow chart.

A retrospective cross-sectional study was conducted at the Kunshan Hospital affiliated with Jiangsu University, utilizing patient data extracted from electronic medical records from January 2017 to August 2023. The study included a total of 4782 patients diagnosed with OPF who required further inpatient surgical treatment. Within three days of admission, these patients underwent blood tests to assess liver and kidney function, as well as lipid and biochemical analyses. OP diagnosis was made on the following inclusion criteria (1): evidence of bone instability and fractures in the absence of other metabolic bone diseases, with physiological bone mineral density (BMD) assessed using T-scores, and (2) OP confirmed by a T-score of −2.5 or less, even in the absence of a predominant fracture (14). The OPF was defined as a fracture resulting from known osteoporosis and/or insufficient trauma, including falls from standing height, lifting, or spontaneous fractures without external trauma (15). Participants were excluded based on the following criteria (1): presence of shock or infection (2), lack of ApoA1 data (3), lack of SUA data (4), history of kidney disease and gout (5), use of statins during hospitalization (6), missing covariate data. A total of 2108 participants were finally included for further analysis (**Figure 1**). This study was approved by the Kunshan Hospital affiliated with Jiangsu University (approval No. 2024-03-053-H00-K01) and complied with the Helsinki Declaration. Personal information was anonymized during data collection and analysis.

### Dependent variable and independent variable

In this study, serum ApoA1 levels were assessed as the independent variable through an electro-chemiluminescence immunoassay conducted on the LABOSPECT 008AS platform (Hitachi High-Tech Co., Tokyo, Japan). The SUA concentration is the outcome variable of this study, measured by an enzymatic colorimetric method. Clinical parameters were measured using the same instruments and by experienced operators following standardized procedures. Prior to participant examinations each day, the machines underwent standard quality control procedures.

### Covariates

This study included various potential covariates, such as age, gender, body mass index (BMI), primary diagnosis, Charlson Comorbidity Index (CCI) (16), hypertension, diabetes, alanine aminotransferase (ALT), aspartate aminotransferase (AST), creatinine(Cr), and albumin. The CCI is a validated measure that quantifies comorbidity burden by assigning weighted scores to 19 different chronic conditions, with higher scores indicating greater comorbidity and increased mortality risk (17). All blood samples were collected on the first morning after admission, following a standardized fasting period of 8-10 hours.

### Statistical analyses

Population demographics, laboratory, and clinical data are presented using standard deviation (SD) or median (25th, 75th percentile) for continuous variables, while categorical data are shown as frequencies (percentages). For univariate analysis of categorical data, Pearson’s chi-squared test or Fisher’s exact test is employed, and for continuous data, independent sample t-tests and Mann-Whitney U tests are used for normally distributed and non-normally distributed data, respectively. Additionally, to explore the relationship between characteristics of OPF patients and uric acid, corresponding univariate analyses were conducted.

The general linear models with appropriate adjustment for covariates were used to investigate the independent relationship between ApoA1 and SUA in OPF. The generated models underwent full calibration (Model 4), partial calibration (Models 2 and 3), and no calibration (Model 1) to explore this relationship. Based on the evaluation of variance inflation factor (VIF) data, covariances were adjusted as follows: Standard 1: The impact of introducing covariates into the basic model or removing covariates from the complete model on the regression coefficient of X is greater than 10%.Standard 2: The P-value of the regression coefficient between Standard 1 or covariate and Y is less than 0.1 (18). Four models were ultimately established: Model 1 without adjustments; Model 2 adjusted for gender, age, BMI, primary diagnosis, and CCI; Model 3 adjusted for gender, age, BMI, primary diagnosis, CCI, hypertension, and diabetes; Model 4 adjusted for gender, age, BMI, primary diagnosis, CCI, hypertension, diabetes, ALT, AST, CR, and albumin.

In addition to linear models, we employed Generalized Additive Models (GAM) to examine the nonlinear relationship between ApoA1 and SUA concentrations. Segmented regression (also known as piece-wise regression) was used to identify threshold effects in the smoothed curves, and the algorithmic approach of maximum likelihood modeling was utilized to recursively compute the inflection points of these unique rate curves (19). After categorizing patients based on specific covariates, additional analyses were conducted to assess the reliability of study results and compare differences among different patient groups. Likelihood ratio tests (LRT) were employed to evaluate interactions and modifications among subgroups (**Supplementary Figure S1**). Additionally, multiple imputation analysis was performed to determine whether the incorporation of covariates for missing data introduced bias into our results (20). The results of the general linear models based on data obtained from multiple imputations are presented in **Supplementary Table S1**.

All analyses were conducted using R packages (http://www.R-project.org, The R Foundation) and EmpowerStats software (http://www.empowerstats.com, X&Y Solutions, Inc, MA, USA) with a significance level set at a two-sided P < 0.05.

## Results

### Patient characteristics

**Table 1** summarizes the baseline characteristics of 2108 OPF patients admitted between January 2017 and August 2023, categorized by quartiles of ApoA1. The patients had a mean age of 68.90 ± 11.14 years, with a gender distribution of 29.08% male and 70.92% female., The average ApoA1 level was 1.21 ± 0.24 mg/dL, and the average SUA level of 283.02 ± 89.86 µmol/L. Patients were stratified into quartiles based on their ApoA1 levels (<1.03, 1.04-1.17, 1.18-1.33, 1.34-2.49 mg/dL), revealing significant differences in Cr, albumin, SUA, and ApoA1 levels.

**Table 1:** Patient characteristics based on ApoA1 quartiles.

Characteristics	Total	Mean ± SD / N (%)				*P*-value	*P*-value*
		Q1(<1.03g/L)	Q2(1.04-1.17g/L)	Q3(1.18-1.33g/L)	Q4(1.34-2.49g/L)		
N	2108	456	546	543	563		
Age, years	68.85 ± 10.99	68.42 ± 11.36	69.06 ± 10.87	68.27 ± 10.82	69.55 ± 10.94	0.193	0.191
BMI, kg/m^2^	23.30 ± 3.25	23.22 ± 3.30	23.32 ± 3.28	23.44 ± 3.19	23.21 ± 3.25	0.636	0.292
ALT, U/L	22.94 ± 21.26	22.73 ± 16.89	23.25 ± 30.92	23.43 ± 18.60	22.34 ± 14.21	0.828	0.439
AST, U/L	25.41 ± 17.18	27.31 ± 26.38	24.93 ± 15.79	24.91 ± 13.33	24.82 ± 11.26	0.069	0.245
CR, umol/L	64.88 ± 34.73	73.11 ± 47.83	66.23 ± 21.43	62.16 ± 22.71	59.54 ± 40.43	<0.001	<0.001
Albumin, g/L	39.91 ± 4.07	37.48 ± 4.61	39.71 ± 3.68	40.56 ± 3.71	41.45 ± 3.30	<0.001	<0.001
SUA, umol/L	283.05 ± 90.50	299.51 ± 95.74	285.46 ± 89.75	277.17 ± 86.63	273.04 ± 88.75	<0.001	<0.001
Apolipoprotein A1,g/L	1.21 ± 0.24	0.92 ± 0.11	1.11 ± 0.04	1.25 ± 0.04	1.52 ± 0.17	<0.001	<0.001
Gender, N (%)						0.685	-
female	1495 (70.92%)	317 (69.52%)	381 (69.78%)	392 (72.19%)	405 (71.94%)		
male	613 (29.08%)	139 (30.48%)	165 (30.22%)	151 (27.81%)	158 (28.06%)		
Primary diagnosis, N (%)						0.629	-
Thoracic vertebral fracture	320 (15.18%)	61 (13.38%)	84 (15.38%)	88 (16.21%)	87 (15.45%)		
Lumbar fracture	590 (27.99%)	116 (25.44%)	166 (30.40%)	148 (27.26%)	160 (28.42%)		
Humerus fracture	194 (9.20%)	44 (9.65%)	40 (7.33%)	58 (10.68%)	52 (9.24%)		
Radial fracture	317 (15.04%)	76 (16.67%)	85 (15.57%)	76 (14.00%)	80 (14.21%)		
Femoral fracture	687 (32.59%)	159 (34.87%)	171 (31.32%)	173 (31.86%)	184 (32.68%)		
CCI score categorical						0.881	-
0	1859 (88.19%)	401 (87.94%)	474 (86.81%)	484 (89.13%)	500 (88.81%)		
1	185 (8.78%)	40 (8.77%)	56 (10.26%)	43 (7.92%)	46 (8.17%)		
≥2	64 (3.04%)	15 (3.29%)	16 (2.93%)	16 (2.95%)	17 (3.02%)		
Hypertension, N (%)						0.038	
NO	1818 (86.24%)	382 (83.77%)	463 (84.80%)	469 (86.37%)	504 (89.52%)		
Yes	290 (13.76%)	74 (16.23%)	83 (15.20%)	74 (13.63%)	59 (10.48%)		
Diabetes, N (%)						0.357	
No	2012 (95.45%)	439 (96.27%)	516 (94.51%)	523 (96.32%)	534 (94.85%)		
Yes	96 (4.55%)	17 (3.73%)	30 (5.49%)	20 (3.68%)	29 (5.15%)		

ApoA1, Apolipoprotein A1; SD, standard deviation; Q1, first quartile; Q2, second quartile; Q3, third quartile; Q4, fourth quartile; BMI, body mass index; ALT, alanine aminotransferase; AST, aspartate aminotransferase; CR, creatinine; SUA, serum uric acid; CCI, Charlson comorbidity index.

P-value*: Kruskal Wallis Rank Test for continuous variables, Fisher Exact for categorical variables with Expects < 10.

### Univariate analyses of factors associated with SUA

**Table 2** presents the results of the univariate analysis, indicating a negative correlation between ApoA1 levels and SUA. In contrast, ALT, AST, albumin, and creatinine exhibited a positive correlation with uric acid levels.

**Table 2:** Univariate analysis for SUA.

Characteristics	Mean ± SD / N (%)	β^a^ (95% CI) *P*-value
Age, years	68.85 ± 10.99	-0.08 (-0.43, 0.27) 0.6549
BMI, kg/m^2^	23.30 ± 3.25	-0.39 (-1.58, 0.80) 0.5221
ALT, U/L	22.94 ± 21.26	0.49 (0.31, 0.67) <0.0001
AST, U/L	25.41 ± 17.18	0.48 (0.26, 0.71) <0.0001
Albumin, g/L	39.91 ± 4.07	1.66 (0.71, 2.61) 0.0006
CR, umol/L	64.88 ± 34.73	0.94 (0.84, 1.04) <0.0001
Gender		
female	1495 (70.92%)	Reference
male	613 (29.08%)	0.77 (-7.73, 9.28) 0.8585
Primary diagnosis, N (%)		
Thoracic vertebral fracture	320 (15.18%)	Reference
Lumbar fracture	590 (27.99%)	0.69 (-11.63, 13.01) 0.9124
Humerus fracture	194 (9.20%)	1.20 (-14.95, 17.35) 0.8839
Radial fracture	317 (15.04%)	1.50 (-12.57, 15.56) 0.8350
Femoral fracture	687 (32.59%)	-3.57 (-15.58, 8.45) 0.5608
CCI score categorical		
0	1859 (88.19%)	Reference
1	185 (8.78%)	3.90 (-9.78, 17.57) 0.5768
≥2	64 (3.04%)	-8.22 (-30.77, 14.34) 0.4752
Hypertension, N (%)		
No	1818 (86.24%)	Reference
Yes	290 (13.76%)	-3.14 (-14.36, 8.07) 0.5829
Diabetes, N (%)		
No	2012 (95.45%)	Reference
Yes	96 (4.55%)	-9.43 (-27.96, 9.10) 0.3185
ApoA1^b^, g/L	1.21 ± 0.24	-30.89 (-46.98, -14.81) 0.0002
ApoA1 quartile, N (%)		
Q1(<1.05g/L)	456 (21.63%)	Reference
Q2(1.06-1.19g/L)	546 (25.90%)	-14.05 (-25.25, -2.86) 0.0140
Q3(1.20-1.34g/L)	543 (25.76%)	-22.34 (-33.55, -11.13) <0.0001
Q4(1.35-2.49g/L)	563 (26.71%)	-26.47 (-37.59, -15.35) <0.0001

ApoA1, Apolipoprotein A1; SD, standard deviation; CI, confidence interval; Q1, first quartile; Q2, second quartile; Q3, third quartile; Q4, fourth quartile; BMI, body mass index; ALT, alanine aminotransferase; AST, aspartate aminotransferase; CR, creatinine; SUA, serum uric acid; CCI, Charlson comorbidity index.

^a^ Dependent variable UA, as a result of univariate analyses for UA.

^b^ For continuous variables.

### Exploration of the association between apoA1 levels and SUA

A univariate linear regression model was used to assess the association between ApoA1 and SUA. The corresponding results for the four models are presented in **Table 3**. In the unadjusted Model 1, a noteworthy correlation was observed between these factors (β = -30.89, 95% CI -46.98, -14.81, P = 0.0002). Adjusted Model 2 (adjusting for age, gender, Body Mass Index (BMI), primary diagnosis, CCI) showed a similar association (β = -30.79, 95% CI -46.93, -14.66, P = 0.0002). Model 3 also demonstrated a significant negative correlation, further adjusting for hypertension and diabetes variables (β = -30.75, 95% CI -46.92, -14.57, P = 0.0002). Even after additional adjustments for ALT, AST, CR, and albumin covariates in the fully adjusted Model 4, the association between the two remained significant (β = -29.25, 95% CI: -45.16, -13.33, P = 0.0003).

**Table 3:** Association between ApoA1 concentrations and SUA level across various models (N=2108).

	Model 1^a^ β(95%CI) *P*-value	Model 2^b^ β(95%CI) *P*-value	Model3^c^ β(95%CI) *P*-value	Model4^d^ β(95%CI) *P*-value
ApoA1 Per 1g/L increment	-30.89 (-46.98, -14.81) 0.0002	-30.79 (-46.93, -14.66) 0.0002	-30.75 (-46.92, -14.57) 0.0002	-29.25 (-45.16, -13.33) 0.0003
ApoA1 quartile				
Q1 (<1.03g/L)	Reference	Reference	Reference	Reference
Q2 (1.04-1.17g/L)	-14.05 (-25.25, -2.86) 0.0140	-14.30 (-25.53, -3.06) 0.0127	-14.18 (-25.43, -2.94) 0.0135	-14.85 (-25.40, -4.30) 0.0059
Q3 (1.18-1.33g/L)	-22.34 (-33.55, -11.13) <0.0001	-22.36 (-33.60, -11.12) <0.0001	-22.41 (-33.66, -11.17) <0.0001	-22.15 (-32.91, -11.38) <0.0001
Q4 (1.34-2.49g/L)	-26.47 (-37.59, -15.35) <0.0001	-26.58 (-37.73, -15.42) <0.0001	-26.57 (-37.75, -15.38) <0.0001	-26.25 (-37.23, -15.27) <0.0001
*P*-value for trend	<0.0010	<0.0010	<0.0010	<0.0010

ApoA1 Apolipoprotein A1, SD standard deviation, CI confidence interval, Q1 first quartile, Q2 second quartile, Q3 third quartile, Q4 fourth quartile, BMI body mass index, ALT alanine aminotransferase, AST aspartate aminotransferase, CR creatinine, SUA serum uric acid, CCI Charlson comorbidity index.

a: No adjustment.

b: adjusted for: gender, age, BMI, primary diagnosis, CCI.

c: adjusted for: gender, age, BMI, primary diagnosis, CCI, hypertension, diabetes.

d: adjusted for: gender, age, BMI, primary diagnosis, CCI, hypertension, diabetes , ALT, AST, CR, Albumin.

Additionally, we conducted a sensitivity analysis where ApoA1 was treated as a categorical variable (quartiles). In the fully adjusted Model 4, the trend of association between ApoA1 and SUA remained consistent (p for the trend was <0.001). To validate the robustness of our findings, we conducted subgroup analyses on various categorical variables. Notably, there was a significant interaction effects between gender and the primary diagnosis observed concerning the association of ApoA1 with UA (**Supplementary Figure S1**).

### Spline smoothing plot and threshold analyses

**Figure 2:**
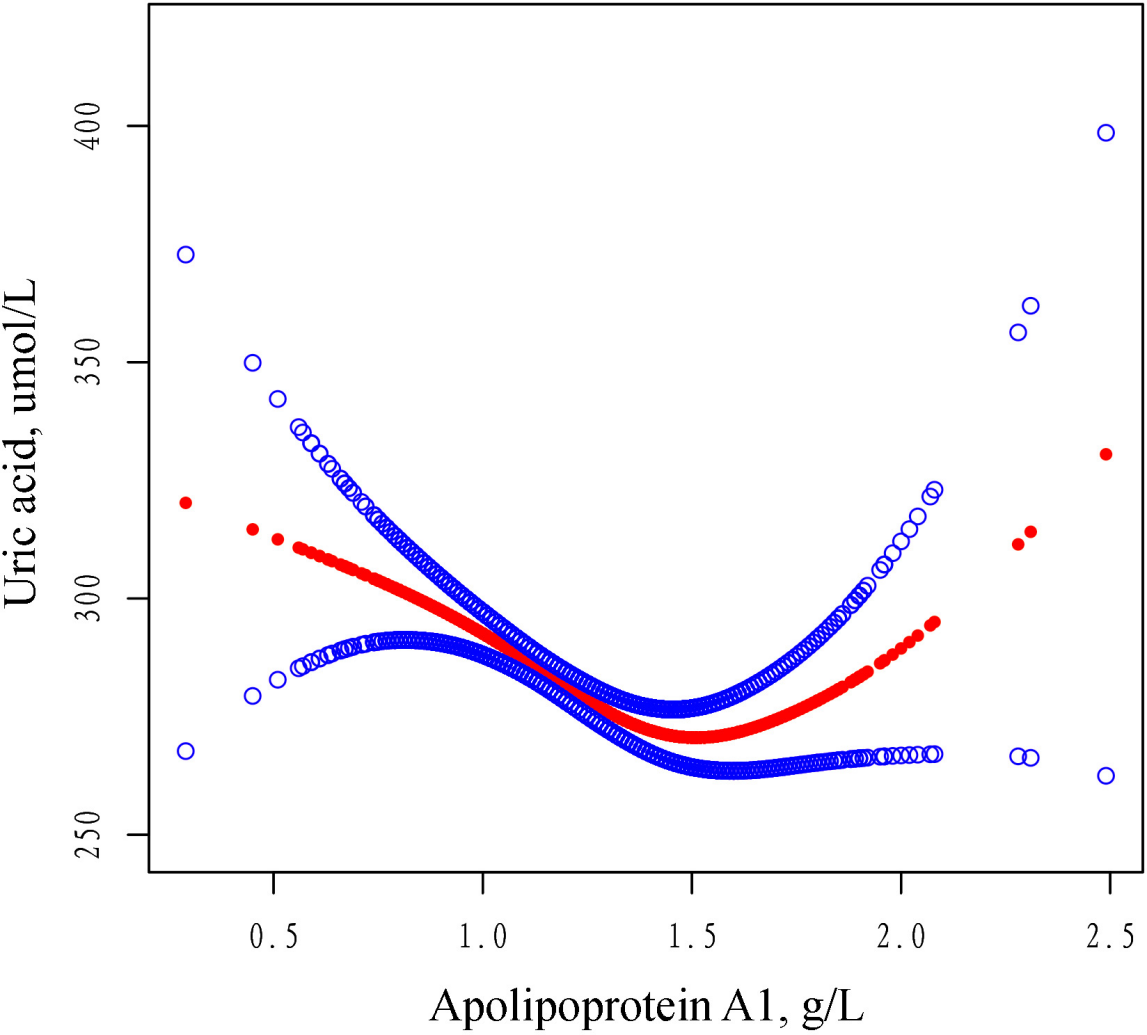
The adjusted smoothed curves illustrate the association between serum ApoA1 levels and the SUA. A generalized additive model identified a threshold non-linear association between serum ApoA1 levels and SUA in patients with OPF. The upper and lower blue curves denote the boundaries of the 95% confidence interval, whereas the central red curve elucidates the association between ApoA1 levels and SUA. The model has been adjusted for variables including gender, age, BMI, primary diagnosis, CCI, hypertension, diabetes, ALT, AST, CR and Albumin. In Model 4, the red curve indicates an inflection point (K) at 1.52 g/L. ApoA1 Apolipoprotein A1, SD standard deviation, CI confidence interval, BMI body mass index, ALT alanine aminotransferase, AST aspartate aminotransferase, CR creatinine, SUA serum uric acid, CCI Charlson comorbidity index.

In this study, we analyzed the nonlinear relationship between ApoA1 and SUA as both were treated as continuous variables (**Figure 2**). The results revealed a threshold nonlinear relationship between ApoA1 and SUA in the study population (adjusted for age, gender, primary diagnosis, BMI, CCI, hypertension, diabetes, ALT, AST, CR, albumin). The segmented regression method was used to determine the inflection point, and we calculated the inflection point to be 1.52 g/L. On the left side of the inflection point, the effect size, 95% CI, and P were -55.20, -75.57 to -34.82, and <0.0001, respectively. Conversely, to the right of the inflection point, the effect size, 95% CI, and P were 77.33, 22.48 to 132.19, and 0.0058, respectively (**Table 4**).

**Table 4:** Threshold effect analysis of the association between ApoA1 and SUA (N=2108).

	Model4^a^	
	β (95%CI)	*P*-value
Model A^b^		
One line effect	-29.25 (-45.16, -13.33)	0.0003
Mode B^c^		
ApoA1 turning point (K), g/L	1.52	
< K	-55.20 (-75.57, -34.82)	<0.0001
> K	77.33 (22.48, 132.19)	0.0058
Slope 2–Slope 1	77.33 (22.48, 132.19)	0.0058
LRT test^d^	<0.0010	

ApoA1 Apolipoprotein A1, CI confidence interval, BMI body mass index, ALT alanine aminotransferase, AST aspartate aminotransferase, CR creatinine, SUA serum uric acid, CCI Charlson comorbidity index.

a Adjusted for Gender, age, BMI, primary diagnosis, CCI, hypertension, diabetes, ALT, AST, CR, albumin.

b Linear analysis, *P*-value < 0.05 indicates a linear relationship.

c Non-linear analysis.

d LRT, likelihood ratio test, *P*-value < 0.05 means Model II is significantly different from Model I, which indicates a non-linear relationship.

## Discussion

To our knowledge, this cross-sectional study is the first to elucidate the potential impact of ApoA1 concentration on SUA levels in hospitalized patients with OPF requiring surgery. Analyzing clinical data from 2,108 patients at Kunshan Hospital affiliated with Jiangsu University, we observed a U-shaped correlation between ApoA1 concentration and SUA levels. Specifically, ApoA1 levels below 1.52 g/L were negatively correlated with SUA concentrations, whereas levels above this threshold exhibited a positive correlation. Notably, the association of ApoA1 with SUA diverges from findings in other studies, suggesting a potentially key role for ApoA1 in uric acid metabolism. This finding advances our understanding of the relationship between ApoA1 and SUA levels. In clinical practice, when ApoA1 levels approach a specific threshold, it may correspond with lower uric acid levels in patients. Therefore, monitoring SUA levels may be warranted when ApoA1 concentrations are either excessively high or low.

Previous studies have predominantly examined the relationship between uric acid levels and lipid profiles. A cross-sectional analysis utilizing data from the US National Health and Nutrition Examination Survey (NHANES) revealed significant inverse associations between SUA levels and both triglyceride and high-density lipoprotein cholesterol (HDL-C) levels (21). Another study, after adjusting for confounders, identified a nonlinear positive correlation between the triglyceride-to-HDL-C ratio (TG/HDL-C) and SUA levels (22). Recent research has reported a negative correlation between elevated SUA and ApoA1 among both atrial fibrillation patients and healthy Chinese individuals (13, 23), suggesting that hyperuricemia may contribute to cardiovascular disease progression by lowering ApoA1. These findings differ from those of the present study, likely due to differences in the study populations. The relationship between serum uric acid and bone metabolism appears to vary according to the specific circumstances. Xiao et al. (24) identified a positive correlation between SUA levels and lumbar spine bone mineral density (BMD) in Han Chinese men over 50 years old. Xu et al. (8) also observed an independent positive correlation between SUA levels and BMD, indicating that uric acid may have a protective effect on bone density in certain circumstances. This protective effect may be attributed to the antioxidant properties of uric acid, which can mitigate oxidative stress that promotes osteoclast activation and bone resorption (25). Conversely, at serum uric acid levels that exceed the physiological threshold (6.8 mg/dL), bone resorption resulting from the formation of monosodium urate crystals caused by excess uric acid has been observed, triggering inflammatory cascades and activating the RANKL/RANK pathway, thereby promoting osteoclast formation and inhibiting osteoblast function (26). Sun et al. (27) identified a close association between higher ApoA1 levels and an increased risk of osteoporosis. Furthermore, experimental studies in animal models have demonstrated that ApoA1 deficiency impairs the differentiation of bone progenitor cells, resulting in enhanced adipogenesis and reduced osteoblastogenesis (28). Consequently, the relationship between ApoA1, SUA, and osteoporosis is intricate and multifaceted. Our study elucidated a U-shaped relationship between ApoA1 and uric acid in OPF patients, a discovery that diverges from the majority of reported literature. This research further enriches the literature on the relationship between apolipoprotein and uric acid levels in the OPF population.

In the OPF population, a U-shaped relationship between ApoA1 and SUA levels was observed, allowing us to identify the inflection point of this association. Based on these findings, we introduce the concept of a “SUA trough” in this population, suggesting that at specific ApoA1 concentrations, particularly around 1.52 g/L, patients may exhibit lower SUA levels. In contrast, SUA levels tend to rise when ApoA1 levels deviate significantly from this point, either too low or too high. This U-shaped relationship suggests that a more nuanced approach to lipid management might be necessary in patients with OPF, where the retention of ApoA1 concentrations around 1.52 g/L may be beneficial for maintaining lower SUA levels. Clinically, this implies that in evaluating the lipid profile of osteoporotic fracture patients, particularly when ApoA1 levels vary markedly from 1.5 g/L, practitioners should also monitor SUA levels. Future studies could investigate the mechanisms by which ApoA1 influences uric acid metabolism and assess whether regulating ApoA1 levels might aid in managing SUA in osteoporotic fracture patients, potentially contributing to optimized long-term outcomes. Our findings provide empirical support for the intricate relationship between lipid and SUA metabolism. Additionally, we conducted exploratory subgroup analyses examining the association between ApoA1 and SUA, which revealed significant interactions with gender and primary diagnosis. These interactions, not previously reported, underscore the need for further investigation into the underlying mechanisms in future studies.

ApoA1, a major component of high-density lipoprotein cholesterol (HDL-C) (29), is inversely correlated with plasma triglycerides in certain conditions, likely due to lipid exchange, hepatic signaling, regulation by microRNA-33, and the role of ATP-binding cassette transporter A1 (ABCA1) in the liver (30). In early animal studies, infusion of purified ApoA1 from healthy human plasma into atherosclerotic rabbits on a high-fat diet significantly reduced atherosclerotic plaque area, lowered serum triglycerides, and reduced low-density lipoprotein cholesterol levels (31). A mechanistic study showed that ApoA1 could modulate cellular metabolism through the AMP-activated protein kinase (AMPK)-mammalian target of rapamycin (mTOR) signaling pathway, which plays a crucial role in regulating both lipid metabolism and uric acid production (32). At optimal levels, ApoA1 maintains metabolic homeostasis by promoting AMPK activation, while either too little or too much ApoA1 can disrupt this balance (33). This provides a potential mechanistic explanation for the inflection point observed in the U-shaped relationship. Recent findings indicate that hepatic fatty acid β-oxidation can activate hypoxia-inducible factor-1α (HIF-1α), which transcriptionally upregulates xanthine dehydrogenase (XDH) and cytosolic 5’-nucleotidase-II (NT5C2) in the uric acid synthesis pathway, thereby promoting hepatic SUA production (34). This evidence suggests that hyperlipidemia could trigger hyperuricemia, highlighting the interlinked roles of lipid and purine metabolism pathways in metabolic disorders. Consequently, ApoA1 may indirectly influence uric acid production through the triglyceride metabolism pathway. In the kidneys, SUA is extensively filtered by the glomeruli and actively transported in the proximal tubules for secretion and reabsorption. Some studies have observed the anti-inflammatory effects of ApoA1 (35). For example, it may protect against diabetic nephropathy by reducing renal inflammation and shielding kidney cells from oxidative damage (36). Additionally, the intestine plays a major role in extra-renal uric acid excretion, with approximately 30% of SUA secreted through the intestinal epithelium and subsequently degraded by gut microbiota. Certain symbiotic flagellated bacteria can stimulate hepatic ApoA1 and HDL production by activating Toll-like receptor 5 (TLR5) in liver cells (37). Moreover, some conserved genes in intestinal microbes have been identified as potential regulators of gut and systemic purine levels (38), indicating that the gut microbiota may mediate the association identified in the present study. While these studies provide mechanistic insights into the association between ApoA1 and SUA, the precise details of the ApoA1-SUA interaction require further exploration.

Our study has several strengths. First, we conducted this research within the osteoporotic fracture population, which differs from previous observations of the inverse relationship between ApoA1 and uric acid, thus broadening our understanding of their relationship. Second, we evaluated the relationship between ApoA1 and uric acid using multiple models and identified a non-linear relationship. With a larger sample size compared to previous literature, we evaluated the association between ApoA1 and uric acid in OPF patients. Additionally, we employed multiple imputation and subgroup analyses for additional exploration, providing potential insights for future patient uric acid metabolism regulation. However, this study has limitations. First, as an observational study, the identified non-linear correlation between apolipoproteins and uric acid should not be interpreted as causal. Second, apolipoprotein levels were measured at a single time point, without considering potential trajectories. SUA levels are influenced by numerous factors, and while we excluded some individuals, there may still be unaccounted factors. Lastly, despite controlling for numerous potential confounders, including demographic variables, chronic conditions, and clinical indicators, information on several possible confounders was not available in the database, and we were thus unable to account for lifestyle behaviors (such as smoking status, alcohol consumption, and dietary patterns) and medication history (particularly the use of diuretics and uricosuric agents) that could have potentially influenced both ApoA1 and SUA levels. Therefore, residual confounding from these unmeasured variables should be considered when interpreting the findings.

## Conclusions

In this study, we identified for the first time a U-shaped relationship between ApoA1 and SUA levels in patients with OPF. Specifically, ApoA1 levels below 1.52 g/L show a negative correlation with SUA, while levels above 1.52 g/L are positively correlated with SUA. This finding addresses a knowledge gap in this area, leading us to propose the novel concept of a “uric acid trough” at an ApoA1 concentration of approximately 1.52 g/L. This suggests that both markedly low and high levels of ApoA1 may adversely impact uric acid metabolism. Further research in a larger patient cohort is warranted to confirm and expand upon these findings.

## Data Availability

The original contributions presented in the study are included in the article/**Supplementary Material**. Further inquiries can be directed to the corresponding authors.

## Supplementary material

Supplementary Figure 1 Effect size of ApoA1 on SUA in prespecified and exploratory subgroups.
