# Supplementary figures and images for: U-Shaped association between apolipoprotein A1 and serum uric acid levels in patients with osteoporotic fractures: a cross-sectional study
Source: Front Endocrinol (Lausanne). 2025 Apr 22;16:1540879. doi: 10.3389/fendo.2025.1540879 (PMC12052554; doi:10.3389/fendo.2025.1540879)

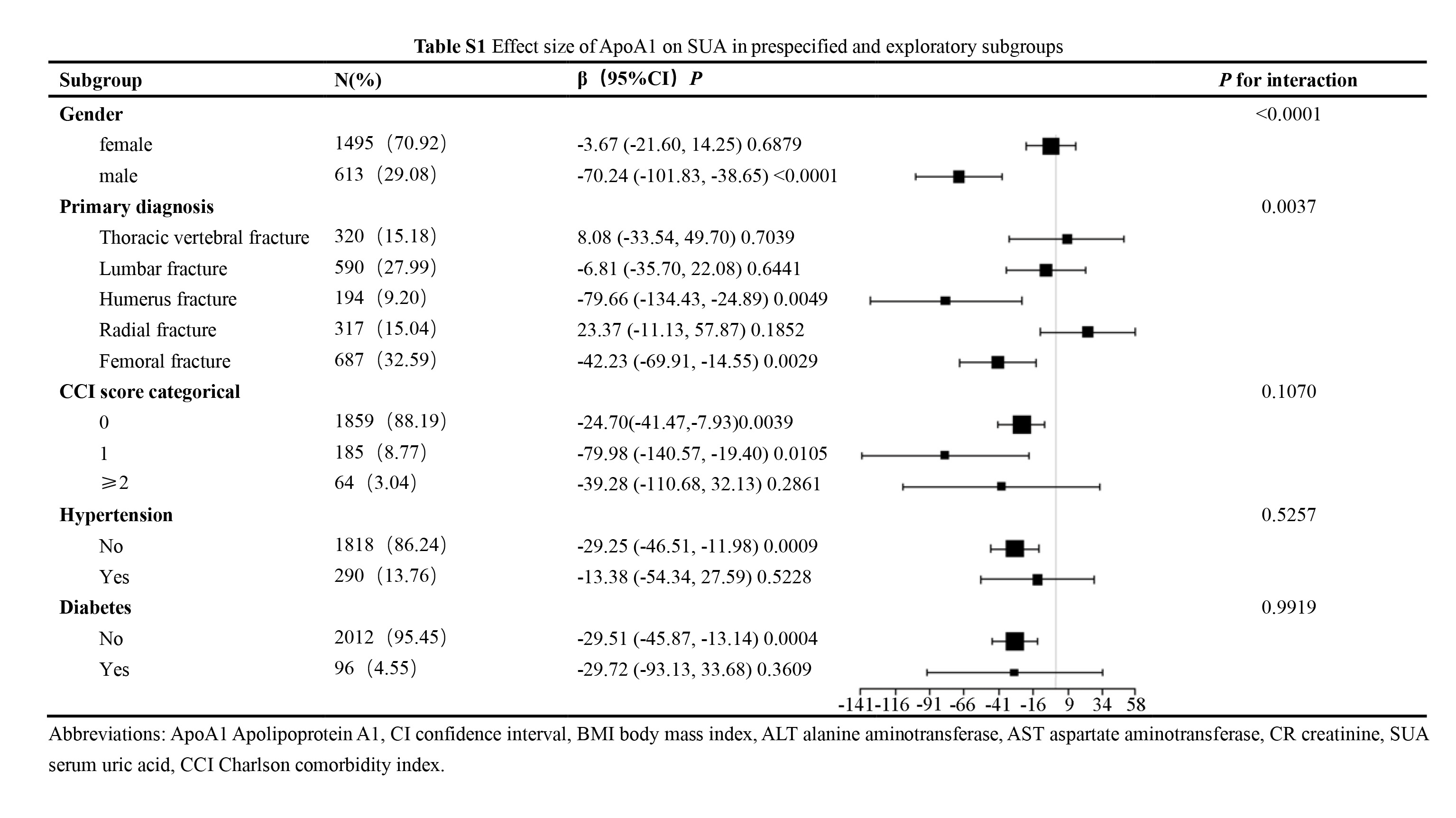

Supplement: Supplementary Figure 1 — Effect size of ApoA1 on SUA in prespecified and exploratory subgroups. [file Image1.jpg]
