# Supplementary material for: U-Shaped association between apolipoprotein A1 and serum uric acid levels in patients with osteoporotic fractures: a cross-sectional study
Source: Front Endocrinol (Lausanne). 2025 Apr 22;16:1540879. doi: 10.3389/fendo.2025.1540879 (PMC12052554; doi:10.3389/fendo.2025.1540879)
Supplement: Supplementary file 3 [file Table2.docx]

**Table S2** Association between ApoA1 concentrations and SUA level across various models with imputed variables from multiple imputation(N=2407)

|  | **Model 1^a^**  **β(95%CI) *P*-value** | **Model 2^b^**  **β(95%CI) *P*-value** | **Model3^c^**  **β(95%CI) *P*-value** | **Model4^d^**  **β(95%CI) *P*-value** |
| --- | --- | --- | --- | --- |
| ApoA1 Per 1g/L increment | -31.5100 (-46.25, -16.77) 0.000028 | -31.26 (-46.05, -16.46) 0.000035 | -31.38 (-46.19, -16.57) 0.000033 | -30.98 (-45.69, -16.26)  0.000037 |

Abbreviations: ApoA1 Apolipoprotein A1, SD standard deviation, CI confidence interval, BMI body mass index, ALT alanine aminotransferase, AST aspartate aminotransferase, CR creatinine, SUA serum uric acid, CCI Charlson comorbidity index

a: No adjustment
b: adjusted for: gender, age, BMI, primary diagnosis, CCI
c: adjusted for: gender, age, BMI, primary diagnosis, CCI, hypertension, diabetes,

d: adjusted for: gender, age, BMI, primary diagnosis, CCI, hypertension, diabetes , ALT, AST, CR, albumin
